# Evaluation of Web-Based Continuing Professional Development Courses: Aggregate Mixed-Methods Model

**DOI:** 10.2196/mededu.7480

**Published:** 2017-10-20

**Authors:** Arezoo Ebn Ahmady, Megan Barker, Myra Fahim, Rosa Dragonetti, Peter Selby

**Affiliations:** ^1^ Nicotine Dependence Service Centre for Addiction and Mental Health Toronto, ON Canada; ^2^ Dalla Lana School of Public Health University of Toronto Toronto, ON Canada; ^3^ Department of Family and Community Medicine University of Toronto Toronto, ON Canada; ^4^ Department of Psychiatry University of Toronto Toronto, ON Canada

**Keywords:** learning, Internet, evaluation studies, tobacco use

## Abstract

**Background:**

Many continuing professional development (CPD) Web-based programs are not explicit about underlying theory and fail to demonstrate impact.

**Objective:**

The aim of this study was to develop and apply an aggregate mixed-methods evaluation model to describe the paradigm, theoretical framework, and methodological approaches used to evaluate a CPD course in tobacco dependence treatment, the Training Enhancement in Applied Cessation Counseling and Health (TEACH) project.

**Methods:**

We evaluated the effectiveness of the 5-week TEACH Web-based Core Course in October 2015. The model of evaluation was derived using a critical realist lens to incorporate a dimension of utilitarian to intuitionist approaches. In addition, we mapped our findings to models described by Fitzpatrick et al, Moore et al, and Kirkpatrick. We used inductive and deductive approaches for thematic analysis of qualitative feedback and dependent samples *t* tests for quantitative analysis.

**Results:**

A total of 59 participants registered for the course, and 48/59 participants (81%) completed all course requirements. Quantitative analysis indicated that TEACH participants reported (1) high ratings (4.55/5, where 5=best/excellent) for instructional content and overall satisfaction of the course (expertise and consumer-oriented approach), (2) a significant increase (*P* ˂.001) in knowledge and skills (objective-oriented approach), and (3) high motivation (78.90% of participants) to change and sustain practice change (management-oriented approach). Through the intuitionist lens, inductive and deductive qualitative thematic analysis highlighted three central themes focused on (1) knowledge acquisition, (2) recommendations to enhance learning for future participants, and (3) plans for practice change in the formative assessment, and five major themes emerged from the summative assessment: (1) learning objectives, (2) interprofessional collaboration, (3) future topics of relevance, (4) overall modification, and (5) overall satisfaction.

**Conclusions:**

In the current aggregate model to evaluate CPD Web-based training, evaluators have been influenced by different paradigms, theoretical lenses, methodological approaches, and data collection methods to address and respond to different needs of stakeholders impacted by the training outcomes.

## Introduction

Web-based courses for busy health care providers (HCPs) allow for iterative knowledge acquisition and application in real-world practice settings at a relatively low cost. A variety of different online tobacco dependence treatment training programs and evaluation methods for HCPs have been used [1]; however, establishing a comprehensive evaluation of the effectiveness of the Web-based program through a dimension of utilitarian to intuitionist remains a challenge. Existing frameworks developed to evaluate classroom-based continuing education trainings are inadequate in evaluating Web-based courses. Evaluation is a necessary component of curriculum design and innovation, assessing the ability to which curricula can meet established benchmarks. However, evaluation design and implementation can also work toward advancing the scholarship of teaching and learning [2]. Despite general consensus on the importance of training and development for increased self-confidence and competence in health care delivery, research suggests insufficient attention is paid to the quality and long-term effect of training [3]. The many evaluation models that have emerged since 1965 range from basic checklists to comprehensive frameworks, aimed at addressing different needs (ie, self, stakeholders, program planners, etc). In order to use an evaluation model effectively, it is necessary to first identify one’s evaluation needs and subsequently determine what is useful in each model [4]. A conceptual framework in its entirety, which may contain a number of tested theories, is neither necessarily a completely tested theory nor is it a linear process [5]. In the absence of a comprehensively tested theory, conceptual frameworks are useful to guide program planners and advance teaching and learning scholarship. The increasing variety of methodological approaches is not only changing the ways in which evaluations are designed and implemented but is also adding rich perspectives to a burgeoning field still too young to settle on any singular, ideal evaluation approach. Evaluators’ preference on paradigm, theoretical lens, methodological approach and methods of data collection leads to different design, data collection, analysis, and interpretation [6]. These divergent visions evaluation resulted in a variety of frameworks used in program evaluation, as they are derived from philosophical and ideological beliefs, different methodological predilection, value assignment, and end user interests.

The goal of this study was to develop a comprehensive, aggregate, and conceptual evaluation framework focusing on the use of paradigm, theory, and methodology for a Web-based training program. The embodiment of a critical realist lens, characterized through a dimension of utilitarian (the greatest good for the greatest number) to intuitionist (the greatest good requires the attention to each individuals benefit) [4], formed the foundational philosophical beliefs of our program evaluation approach. This study pilots this evaluation framework using a Web-based training in tobacco dependence treatment, the Training Enhancement in Applied Cessation Counseling and Health (TEACH) Core Course, to identify the primary factors that guide the TEACH evaluation and to examine the feasibility of its application for researchers, HCPs, and other relevant stakeholders.

## Methods

Over the past decade, the TEACH Project at the Centre for Addiction and Mental Health (CAMH), has become a benchmark training program for health care providers (HCPs) at local, national, and international levels [7]. The TEACH model incorporates all components of the Knowledge-to-Action (KTA) framework to address the wider tobacco epidemic through evidence-based tobacco dependence treatment [8]. As part of the TEACH Project’s Certificate Program in Intensive Cessation Counseling, participants are expected to complete: (1) a 10-hour Web-based prerequisite course, (2) a 19.5-hour Web-based Core Course, and (3) a 13.5-hour Web-based specialty course.

### Developing an Aggregate Model for Evaluation

The TEACH Project based their evaluation approach on Moore et al’s and Kirkpatrick’s frameworks to evaluate continuing professional development (CPD) education [5,7,9,10]. The Moore et al’s evaluation framework, which includes seven levels of training impact to evaluate, is the gold standard in evaluating CPD education. Moore et al’s framework is an ideal approach to measure CPD educational outcomes, assess impact by focusing on the target outcomes of training, and iteratively modify training to achieve the intended results (ie, the evaluator must ask themselves *How will I do it?*) [5,10]. Also, we chose Kirkpatrick’s evaluation framework which outlines four levels of training effectiveness by tracking improvements in participant’s reactions, learning, behavior and results (ie, the evaluator must ask themselves *How will I know when I have done it?*). This framework is one of the most well-known and widely used evaluation models in assessing the effectiveness of training and learning [9]. However, we decided to add on Fitzpatrick’s approach [11] to create an aggregate model in order to discuss, identify, and justify the primary factors that guide or direct our evaluation approach including paradigm, theory, methodology and methodological tools (ie, the evaluator must ask themselves *What am I doing and why am I doing this?*) [11]. Fitzpatrick et al classifies evaluation into four core approaches: (1) comprehensive judgment of the quality of the training, including expertise and consumer/learner-oriented evaluations, (2) characteristics of the training, including objective-oriented evaluations, (3) decisions to be made about the program, including management-oriented evaluations, and (4) participation of stakeholders (including patients, managers, HCPs, and funders) in the program. These four Fitzpatrick categories can respond to differing needs among stakeholders impacted by the evaluation in multiple contexts and can be easily distributed along House’s dimension [12] of utilitarian to intuitionist approaches for program evaluation. Embodying a critical realist lens [6,13] through House’s dimension could support the notion that quantitative and qualitative methodologies are both equally warranted in fulsomely understanding training success and impact.

**Figure 1:**
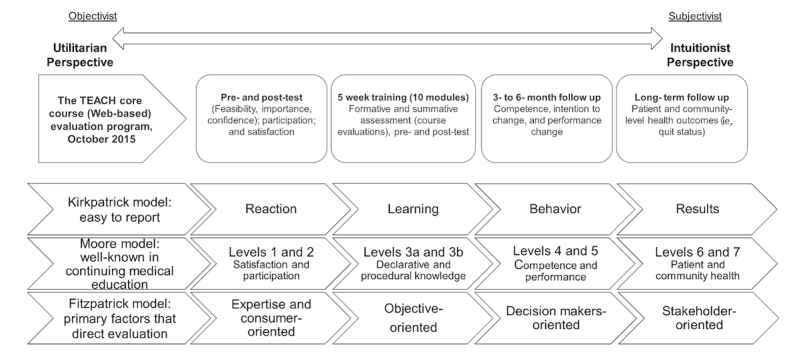
Distribution of four categories of aggregate evaluation approaches on the dimension of utilitarian to intuitionist perspective for the study.

We developed an aggregate evaluation framework (Figure 1), adopted from three conceptual frameworks, Fitzpatrick et al [11], Moore et al [5], and Kirkpatrick [9], to assess a CPD program (the TEACH project) in order to focus on achieving desired outcomes with a critical realist lens through House’s dimension of utilitarian (objectivist ontology) to intuitionist (subjectivist ontology) [4].

Taken together, this aggregate evaluation model can elicit the following primary factors: (1) direct instructional design strategies during educational planning, organizing, implementing, and evaluating, (2) identify how HCPs learn, (3) determine how and where assessment can be used to measure progress of the program, and (4) identify ways to inform decision makers regarding evaluation outcomes.

We examined the aggregate model’s feasibility by evaluating the data which has been collected through one of our cohorts of the TEACH Web-based Core Course in October 2015 with a total of 48 participants. We measured training effectiveness through the following four evaluation approaches, developed based on the aggregate model:

#### Approach 1 (Utilitarian)

Approach 1 is based on Fitzpatrick et al’s expertise and consumer/learner-oriented approaches, Moore et al’s Levels 1 and 2 (Participation and Satisfaction), and Kirkpatrick’s Level 1 (Reaction *)*. This approach focuses on the number of participants (learner-oriented) who completed the training, overall satisfaction, the degree to which participant’s expectations of the quality of the training were met, and quality of the program as compared with other existing programs. We capture participation and satisfaction through the registration database, formative evaluations administered throughout the training, and a summative evaluation administered at the end of the training. We measure quality of the program through evidence of accreditation by an external body. This is aligned with an expertise- and consumer/learned-oriented approach in which participants make valuable judgments based on training credibility (eg, *is the training peer-reviewed, reputable, will I receive a certificate*).

#### Approach 2 (Utilitarian to Intuitionist)

Approach 2 is based on Fitzpatrick et al’s objective-oriented approach, Moore et al’s Levels 3a and 3b (Declarative and Procedural Knowledge), and Kirkpatrick’s Level 2 (Learning). This approach focuses on the impact of training on participants involved (ie, the degree to which participants can (1) state what the training intended them to know and (2) state how to do what the training intended them to know). To achieve this, an evaluator needs to determine the extent to which clearly defined course objectives have been met immediately post course. We capture declarative and procedural knowledge through pre- and postcourse assessments. This approach helps instructional designers, evaluators, and other stakeholders judge the training’s success or shortcomings through some of the trainings immediate outcomes (eg, have knowledge and skills improved among participants post course). We also capture participant learning through the administration of a competency-based exam administered post course. Participants are required to achieve a grade of 70% or higher in order to pass the course. Additionally, participants are able to provide free text comments and open-ended feedback questions for course development to enhance learning for future participants in the formative evaluations administered during the course.

#### Approach 3 (Utilitarian to Intuitionist)

Approach 3 is based on Fitzpatrick et al’s management-oriented approach, Moore et al’s Levels 4 and 5 (Competence and Performance), and Kirkpatrick’s Level 3 (Behavior). The central concern for this approach is to identify and meet the information needs of managerial decision makers, evaluate self-assessed practice change, and implementation of knowledge and skills (ie, the degree to which participants do what the training intended them to do in their practice setting). We measure competence and performance through follow-up surveys administered 3- and 6- months post training. Quantitative data collected 3- and 6- months post training can provide decision makers with evaluation data that demonstrates program effectiveness over a longer period, which can be helpful in guiding decisions for program continuation or expansion. To ensure an intuitionist approach to evaluation, 3- and 6-month surveys should include qualitative response options to capture participants’ experience with practice change post course. This cohort study did not include qualitative response options in the 3- and 6-month follow-up surveys; however, our revised tools will incorporate this approach.

#### Approach 4 (Intuitionist)

Approach 4 is based on Fitzpatrick et al’s stakeholder-oriented approach, Moore et al’s Levels 6 and 7 (Patient and Community Health), and Kirkpatrick’s Level 3 (Results). This approach focuses on a practical participatory evaluation approach, the long-term outcome in job-related performance, and institutional and community level changes as a result of the training program. Patient and community health outcomes are measured through connecting previous evaluation data with the current health outcomes and subsequent linkage with population health data. However, practice change and implementation is dynamic and complex behavior. Additionally, connecting previous evaluation data to patient and community health outcomes is not always easy, feasible, and will only be able to show a marginal effect of the training because of competing factors. Accordingly, a more intuitionist approach rooted in subjectivist epistemology is required for Approach 4. Long-term follow-up through qualitative inductive and/or deductive approaches should involve multiple stakeholders (including administrators, patients, HCPs, and faculty members) in determining the program’s success and shortcomings (ie, by using retrospective post then predesign in-depth-interviews). This cohort study did not include Approach 4 in the protocol; however, our future evaluation research will explore this level.

Figure 1 provides a visual representation of these four evaluation approaches, unequally distributed along House’s dimension of utilitarian to intuitionist evaluation [4,12]. Although the many different approaches to evaluation may appear convoluted, their diversity allows evaluators to pick and choose either the approach or the elements of an approach that will work best for program evaluation.

### Application of the Aggregate Model: Evaluation of the TEACH Web-Based Core Course

A mixed-methods design was undertaken using data collected through formative evaluations administered following each Web-based module and a summative evaluation administered post course, which collected both quantitative and qualitative feedback, pre-and postcourse assessments of knowledge and skills, a competency-based exam administered post course, and 3- and 6-month follow-up surveys measuring self-reported practice change. Accordingly, we were able to pilot Approaches 1 to 3 of our aggregate model. The numeric data were summarized as descriptive statistics using IBM SPSS Statistic 24 for analysis. Simple frequencies and percentages were calculated; additionally, means and standard deviation for the pre- and postcourse assessments were obtained. Paired *t* tests, analysis of variance (ANOVA) and CI were calculated to test the difference between and within the groups with a probability level of .05. Missing data were replaced with grand mean or modal responses for continuous and categorical variables. To gather qualitative data, the formative and summative questionnaires with extensive qualitative comments used thematic analysis to present participant’s evaluation of the course. SPSS 24 and NVivo 11 (QSR International) were used to perform analysis of quantitative and qualitative data. At the time this study was designed, the CAMH Research Ethics Board deemed that formal review and approval was not required for the study.

## Results

### Approach 1

Following expertise-oriented approach, TEACH is accredited by the University of Toronto’s Faculty of Medicine CPD program, as well as the College of Family Physicians of Canada, Royal College of Physicians and Surgeons of Canada, Canadian Addiction Counsellors Certification Federation, and Royal College of Dental Surgeons of Ontario. A total of 59 participants registered for the course, and 48 participants (81%) completed all course requirements. Table 1 shows 42 % (20/48) of the participants were nurses (registered nurses, nurse practitioners, and case manager nurse). Using the utilitarian lens and consumer/learner-oriented approach, the overall satisfaction rating for the course was 4.55/5 (where 5=best/excellent).

### Approach 2

The pre- and postcourse assessments consisting of 29 closed-ended questions related to course competencies were used to collect quantitative data, and paired samples *t* tests were undertaken. Table 2 presents results of quantitative analysis of self-perceived knowledge for each course competency, demonstrating an objective-oriented approach. A Wilcoxon test was used, and the findings indicated a significant difference between self-reported pre- and postknowledge ranks for all course competencies during the TEACH Core Course, *Z*=6.03, *P*<.005.

In addition, we measured participants’ feasibility, importance, and confidence of applying course competency areas, through 12 closed-ended questions. For each domain, a 0-10 Likert-scale (10 being the highest rating) was used. Data normality can be observed in the analysis of feasibility and confidence with the Kolmogorov-Sminrov & Lilliefors test (95% CI). However, data normality was not observed (*P*<.001) for the importance variable. A nonparametric approach was used to support the analysis of the Likert scale responses of feasibility, confidence, and importance (Table 3). Wilcoxon tests of the responses to performance statements about feasibility, importance, and confidence revealed significant differences between self-reported pre- and postlearning at *P* ˂.001.

**Table 1:** Reporting of demographic variables in 48 participants who completed the Training Enhancement in Applied Cessation Counseling and Health (TEACH) Core course in October 2015.

Discipline represented		Participants, n (%)
**Health care professionals**		
	Pharmacist	9 (18.75)
	Nurse	20 (41.67)
**Allied health care professionals**		
	Aboriginal health worker	1 (2.08)
	Addiction counselor	2 (4.17)
	Dental assistant, hygienist, or therapist	1 (2.08)
	Dietitian or nutritionist	1 (2.08)
	Occupational therapist	2 (4.17)
	Respiratory therapist, clinical perfusionist, or asthma educator	1 (2.08)
	Social worker	4 (8.33)
**Health support services**		
	Health promoter/educator	2 (4.17)
	Manager/coordinator	2 (4.17)
	Other	3 (6.25)
**Clinical contact with clients**		
	Yes	42 (87.50)
	No	4 (8.33)
	Unsure	2 (4.17)
**Years providing cessation**		
	1 year or less	16 (33.33)
	2-5	11 (22.92)
	6-10	8 (16.67)
	10+	4 (8.33)
	Never	9 (18.75)
Total number of participants, N		48 (100)

**Table 2:** Wilcoxon test (95% CI) of reaction of health care providers to eight competency domains (pre- and postcourse assessment) through objective-oriented approach (0-10 rating scales, 10 being the highest rating).

Pre-post learning objective	Domain	*Z*	Significance (*P* value)
1	Impact of tobacco use	4.49	<.001
2	Tobacco use assessments	5.86	<.001
3	Motivational interviewing	5.42	<.001
4	Developing a quit plan	5.81	<.001
5	Evidence-based psychosocial interventions	5.85	<.001
6	Evidence-based pharmacological interventions	5.41	<.001
7	Harm reduction approaches	5.43	<.001
8	Relapse prevention strategies	5.86	<.001

**Table 3:** *Z* and *P* values of health care professionals’ self-reported response to feasibility, importance, and confidence in use of the modules before (n=48) and after (n=48) conducting Web-based Training Enhancement in Applied Cessation Counseling and Health (TEACH) core course in October 2015.

Questions	*Z*	*P* value
Feasibility	3.90	<.001
Importance	2.59	<.001
Confidence	5.87	<.001

In order to measure if the participants’ discipline had an effect on their self-reported knowledge and skills post course, a between- and within-group ANOVA was performed. The assumption of normality was evaluated using the Shapiro-Wilk test at a 5% significance level. We found evidence to conclude that self-reported knowledge and skills postcourse assessment scores are normally distributed for HCPs, *W*=.96, *P*=.38, and for Allied HCPs, *W*=.91, *P*=.21. However, in addition, we found evidence to conclude that self-reported knowledge and skills postcourse assessment scores are not normally distributed for Health Support/Research Services, *W*=.65, *P*=.002.

The percentage distribution for the competency-based exam indicates successful achievement of intended outcomes whereby 47 out of 48 participants received a passing grade (n=10 between 80%-90% and n=37 between 90%-100%), with only one participant receiving a failing grade below 70%. Finally, thematic content analysis was conducted for the qualitative comments provided by participants in the formative (34 comments) evaluations which were related to the three themes: (1) *knowledge acquisition*, (2) *recommendations to enhance learning for future participants,* and (3) *plans for practice change* (Table 4). To follow subjectivist epistemology through the intuitionist evaluation concept, the following five themes emerged from 61 qualitative comments in the summative evaluation administered at the end of course: (1) *learning objectives*, (2) *interprofessional collaboration*, (3) *future topics of relevance*, (4) *overall modification,* and (5) *overall satisfaction.*

**Table 4:** Qualitative formative feedback provided by participants of the Training Enhancement in Applied Cessation Counseling and Health (TEACH) cohort core course in October 2015.

Themes	Total coverage	Examples
Knowledge acquisition	18.5%	“Having had no prior experience in tobacco cessation, I learned a great deal from this module. Overall constructive for me.”
		“Difficulty with some material because of lack of knowledge with medications. Noted as an area to spend more time on, personally. I believe the material provided will be beneficial in enhancing my knowledge base.”
Recommendations to enhance learning for future participants	70%	“For questions answered incorrectly, it would be helpful if there was a reference provided so that I could go back and locate where that information to taught.”
		“More examples of case studies for complex clients, when and how to double patch, etc.”
Plans for practice change	11.5%	“I found this module very useful in helping me think of ways in which I can change my practice to include the 5Rs and tobacco cessation discussion for every client at every visit.”
		“Great Module!! There are so many concrete clinical tools that I plan to utilize from this module.”

### Approach 3

Follow-up surveys administered 3- and 6-months post training were analyzed with a management-oriented approach. We evaluated participant’s self-reported implementation of tobacco cessation knowledge and skills, the number of clients seen, the dissemination of program material to other providers, barriers to change, and future intentions to implement knowledge and skill into practice. The average response rate for the 3-month follow-up survey was 27.08% and 29.17% for the 6-month follow-up survey. Responses indicate that at 3-month follow-up, 76.9% of participants were offering individual tobacco cessation sessions with clients, which increased to 85.7% at the time of 6-month follow-up. Participants provided information on what they perceived to be barriers to changing their practice and implementing new cessation programming post course. The need for *more practice* was identified as the major barrier at the 3-month follow-up point. At 6-month follow-up, 64% of respondents still identified the need for more practice as a barrier. Three additional barriers were identified at the 6-month follow-up and included *finding the time* to offer tobacco cessation counseling (an average of 71%), struggling with *patients’ motivation* to attend cessation sessions and *wanting more financial support* for cessation programs and services (50% for each). Another important outcome from using a utilitarian lens through a management–oriented approach relates to the dissemination of tobacco cessation knowledge and skills to other providers. At both follow-up time points, participants were asked whether they had communicated any of the knowledge or skills they had learned to colleagues since completing the course. At the 6-month follow-up time point, 78.90% of participants indicated that they had been involved in informal discussion/information sharing with colleagues; 32.90% indicated that they had offered brief presentations (up to 1 hour) with colleagues; and 8.11% indicated that they had written articles or reports to share with colleagues.

## Discussion

### Principal Findings

Previously, the TEACH project was introduced as the first university-accredited continuing education certificate program in Canada that focused on evidence-based research for intensive cessation training, leading to enhanced system capacity. Study findings suggest that the certificate program impacted clinical practice, highlighting successful knowledge transfer from research to practice [7]. With its focus on a detailed evaluation plan that adds rigor to knowledge translation initiatives, we developed an aggregated evaluation model in which different models of evaluation were grouped according to the similarity of their values, their philosophies, and their methodological approaches. This study demonstrates the feasibility of embodying a critical realist lens, through a dimension of utilitarian to intuitionist evaluation, and use of a mixed-methods approach to design an aggregate model of Fitzpatrick, Moore, and Kirkpatrick for the purpose of quality improvement and to achieve evaluation goals. We then applied this model to evaluate the effectiveness of the TEACH Web-based Core Course, and the results from Approaches 1 to 3 demonstrated that TEACH has been successful in the following: providing Web-based training in cessation counseling to a range of HCPs with different disciplines; high ratings for instructional content and overall satisfaction (expertise and consumer-oriented approaches); a significant increase in participants’ knowledge and skills (objective-oriented approach); and high motivation to change and sustain practice change (management -oriented approach). This model has also helped us to (1) identify the primary factors that guide our program evaluation, (2) balance the importance of utilitarian and intuitionist philosophy in guiding methodological approaches and tools, and (3) encourage the involvement of multiple stakeholders in CPD program evaluation. Our aggregated model classifies the different evaluation approaches influenced by differing views of ontology and epistemology, as well as the methods for obtaining valid information based on what we see as the driving force behind doing the evaluation, and the factors that influence the choice of what to study and the way in which the study is conducted [14].

Approach 1 of our evaluation model directs us to a comprehensive judgment of the quality of the program, which includes expertise-oriented and consumer-oriented evaluation. They are the oldest approaches in evaluation, directing evaluators to focus on valuing or judging the quality of the program they are evaluating [15,16]. Scriven [16] argues that consumer/learner-oriented evaluation factors such as participation and satisfaction can serve as the key criteria for evaluating a program. Participation can elicit some indication whether training is competitive (consumer/learner-oriented), particularly if the training is accredited (expertise-oriented). The consumer/learner-oriented approach in Approach 1 is consistent with House’s [12] conception of utilitarian evaluation to maximize satisfaction among participants. Accordingly, evaluators can focus on total group gains by using outcome scores (eg, satisfaction data). An expertise-oriented approach to evaluation through accreditation is assuring the academic community, the general public, HCPs and other related agencies that a training has been designed using appropriate educational objectives and has the established infrastructure to facilitate participant achievement. This finding is aligned with a previously published study by Kirkwood who emphasized the impact of course accreditation in program evaluation [17].

Approach 2 of our evaluation model helps us to determine the extent to which our training objectives have been achieved. Bloom et al [18] not only emphasize the importance of identifying appropriate objectives in training development for the subject matter but also in developing and measuring participant achievement of these objectives. Cronbach [19,20] also developed an approach to using an objective and associated measurement technique for the purpose of quality improvement in training content, consistent with our objective-oriented approach to evaluation, where the focus is on specifying objectives and determining the extent to which these objectives have been met (ie, measuring changes in knowledge, feasibility, importance and confidence in course competencies). Aligned with intuitionist philosophy, collection of qualitative feedback can also elicit the extent to which training objectives were achieved. For instance, qualitative feedback in this pilot illuminated potential areas for future skill development and comments for improvements.

Approach 3, which is oriented to decision making, focuses on how evaluation outcomes can support decision makers (eg, managers and funders) in making judgments regarding program improvements and continuation. On the basis of a review of studies on commitment to change (that can predict actual change in practice) [21,22], we conducted 3- and 6-month follow-up surveys post training to evaluate practice change. Whereas collecting self-reported practice change data through our current quantitative approach is helpful in identifying implementation of knowledge and skills post course, it does not provide the information needed to guide specific program improvement [5]. Accordingly, our future evaluation design, informed by intuitionist philosophy, will incorporate qualitative questions in the 3- and 6-month follow-up surveys to support decision makers with their ability to make judgments regarding program improvement (management-oriented approach).

The application of the entire aggregate model through Approach 4 has the potential to involve different stakeholders, including those directly impacted by training (eg, patients) rather than leaving decision making of program changes and implementation to training participants and program managers. Future directions of our program evaluation will involve a long-term evaluation of TEACH through administration of in-depth interviews with previous participants, their patients, and their managers in order to fully capture the intuitionist approach to program evaluation. This evaluation will also connect participant previous evaluation data to patient health outcomes in order to determine training impact beyond the participant [12]. This aggregated model has a number of limitations. At various times, policy makers, funding organizations, planners, managers, or HCPs need to distinguish worthwhile training programs from ineffective ones and revise existing ones so as to achieve desirable results. To do this comprehensively, an evaluation approach needs to include cost-benefit analysis measures. Approach of aggregate model has the potential to objectively measure the effectiveness and efficiency of training by adding a question related to time spent completing the course in formative or summative evaluations.

Another limitation of this study was that we performed our pilot study through the Web-based version of the TEACH course in 2015 with 48 participants. This was done to assess the feasibility of the new evaluation model that required a redesign of evaluation questions and approaches. We were consequently unable to utilize our available, large-scaled data from our previous study [7] since the previous evaluation data did not match our newly piloted questions. Furthermore, we did not use a sample size calculation for this study. We tested the aggregate evaluation model with a cohort of participants in 2015 who were the first to experience our new evaluation model. The purpose of the pilot study was to test the feasibility of addressing the four approaches of the new aggregate model and our design. In general, sample size calculations may not be required for some pilot studies [23]. In addition, as argued by Connelly [24], Hill [25], Julious [26] in the medical field, and Van Belle [27], 10 to 30 participants is a sufficient sample size for a pilot study. This pilot study of 48 participants was a good way to troubleshoot our developed aggregated model, familiarize the team with the procedures, and to test for potential flaws in the model and experimental design.

Another limitation of the aggregate model is applied in its entirety (ie, including Approach 4)—the evaluation can be complex and resource intensive. To address this limitation, evaluators need to consider the resources and time they have available and if Approach 4 fits within a feasible scope of work. Also, we achieved 27% and 29% response rates for the 3-month and the 6-month Web-based follow-up surveys despite subsequent mailing reminders to nonrespondents. Although we hoped for a better response, other surveys of HCPs also have tended to generate low response rates [28,29].

Using a mixed-methods approach that balances both quantitative and qualitative data as equally valuable in evaluation is consistent with using a realist approach to identify the contextual factors of successful training programs, since these factors are intimately connected to the success or failure of a training [6]. The quantitative results from our evaluation, are aligned with objectivist epistemology in valuing numerical outcomes to clearly define program outcomes at various time points and demonstrate overall impact of the training program. Similar to many other evaluation approaches, quantitative results can be used to guide evaluators, managers, planners, and participants in distinguishing worthwhile training programs [30] from ineffective ones. One of our evaluation goals will be to improve the response rates to these surveys administered post course. The qualitative results from the TEACH project’s evaluation approach are aligned with subjectivist epistemology in valuing participant feedback in determining program success and impact. Qualitative results can also be used to distinguish worthwhile training programs from ineffective ones and are helpful in guiding program modifications. Our future research will incorporate this more fulsomely through the inclusion of qualitative questions in the 3- and 6-month follow-up surveys and through semistructured in-depth interviews with HCPs, their administrators, and their patients post training.

Implementation models for dynamic human behaviors (ie, practice change), such as the KTA cycle, require a comprehensive evaluation framework with different approaches that can inform future decision making. Some evaluators have lamented the proliferation of evaluation models and urged that an effort be made to synthesize existing models [31]. Conversely, some studies demonstrated that the goal of attaining uniformity in evaluation methods and measures cannot be attained without prematurely inhibiting crucial developments in the field of evaluation [32].

### Conclusions

In this evaluation study, different approaches helped us to comprehend the wide range of needs related to evaluating the Web-based TEACH Core Course. Our predispositions and preferences on philosophical and methodological dimensions led us to choose different models, methodologies, data collection tools, analysis methods, and interpretive techniques. As we move forward, we must identify what is useful in each approach when faced with a specific evaluation need. This study also helped to demonstrate that the aggregate model can detect the effects and impact of Web-based courses because of the richness of data collected in each approach impacting different stakeholders. This comprehensive evaluation approach appears compatible and applicable to other programs based on implementation frameworks (eg, KTA). Some of the key features and major characteristics of the aggregate model are as follows:

- When developing an evaluation model, one must consider paradigm, theoretical framework, methodological approach, and data collection methods.
- A comprehensive evaluation model should include qualitative (deductive or inductive) and quantitative approaches for data collection, analysis, and interpretation.
- Utilitarian approaches to evaluation can be helpful in identifying impact through a more objective lens. However, an intuitionist approach, through a subjectivist lens, can uncover information to guide program improvements, beyond what may have been originally expected.

## Abbreviations

ANOVA: analysis of variance
CAMH: Centre for Addiction and Mental Health
CPD: continuing professional development
HCPs: health care providers
KTA: Knowledge-to-Action
TEACH: Training Enhancement in Applied Cessation Counselling and Health
